# Exercise protects vascular function by countering senescent cells in older adults

**DOI:** 10.3389/fphys.2023.1138162

**Published:** 2023-04-06

**Authors:** Jinqi Meng, Qi Geng, Sheng Jin, Xu Teng, Lin Xiao, Yuming Wu, Danyang Tian

**Affiliations:** ^1^ Department of Sports, Hebei Medical University, Shijiazhuang, China; ^2^ Hebei Key Lab of Laboratory Animal Science, Hebei Medical University, Shijiazhuang, China; ^3^ Department of Physiology, Hebei Medical University, Shijiazhuang, China

**Keywords:** exercise, senescence, vascular function, cardiovascular diseases, aging

## Abstract

Blood vessels are key conduits for the transport of blood and circulating factors. Abnormalities in blood vessels promote cardiovascular disease (CVD), which has become the most common disease as human lifespans extend. Aging itself is not pathogenic; however, the decline of physiological and biological function owing to aging has been linked to CVD. Although aging is a complex phenomenon that has not been comprehensively investigated, there is accumulating evidence that cellular senescence aggravates various pathological changes associated with aging. Emerging evidence shows that approaches that suppress or eliminate cellular senescence preserve vascular function in aging-related CVD. However, most pharmacological therapies for treating age-related CVD are inefficient. Therefore, effective approaches to treat CVD are urgently required. The benefits of exercise for the cardiovascular system have been well documented in basic research and clinical studies; however, the mechanisms and optimal frequency of exercise for promoting cardiovascular health remain unknown. Accordingly, in this review, we have discussed the changes in senescent endothelial cells (ECs) and vascular smooth muscle cells (VSMCs) that occur in the progress of CVD and the roles of physical activity in CVD prevention and treatment.

## 1 Introduction

Blood vessels are conduits for blood and circulating factors such as oxygen, nutrients, and waste products. They primarily consist of two major cell types: endothelial cells (ECs) and vascular smooth muscle cells (VSMCs) (Li et al., 2018), which are essential for homeostasis in mature blood vessels. ECs are a single layer of vulnerable and crucial cells constituting the inner layer of the blood vessel. ECs function as a barrier that separate blood from the vessel wall and regulate the permeability of vessels, helping to prevent thrombosis, inflammation, and atherosclerosis (Jourde-Chiche et al., 2019). VSMCs are highly differentiated cells located in the medial layer of blood vessels; they manifest as a differentiated contractile phenotype in the physiological state and shift to a dedifferentiated synthetic phenotype in response to various stimuli and damage. Alongside VSMCs, the tunica media of blood vessels also contains extracellular matrix (ECM) components (Liu and Gomez, 2019).

Aberrant ECs and VSMCs promote CVD, including atherosclerosis, abdominal aortic aneurysm (AAA), arterial stiffness, hypertension, and heart failure. CVD is the most common disease and the leading cause of morbidity and mortality, especially in the older population (Partridge et al., 2018).

## 2 Characteristics of senescent blood vessels

**FIGURE 1:**
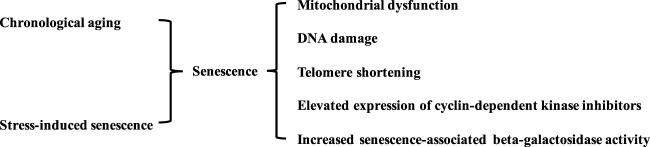
Characteristics of senescent blood vessels. Vascular senescence is induced by various stimulus and biological aging. The senescence in blood vessels is characterized by mitochondrial dysfunction, DNA damage, telomere shortening, elevated expression of cyclin-dependent kinase inhibitors, and increased senescence-associated beta-galactosidase activity.

Life expectancy has increased with advances in modern medicine, higher food quality, vaccinations, and improved housing conditions. However, conflicts have arisen between prolonging lifespan and increased living quality expectancy (Sykorova and Flegr, 2021). Aging is not pathogenic; however, physiological and biological decline during aging is linked to several diseases, including CVD, systemic metabolic disorders, cancer, and neurodegenerative diseases (Li et al., 2021). The biological aging process is characterized by mitochondrial dysfunction, DNA damage, and telomere shortening. Stress-induced premature senescence is another type of cellular senescence. This is triggered by various stimuli, such as oxidative stress, mitogenic stimuli, metabolic stress, and oncogenic activation. Biological aging and stress-induced senescence are complex irreversible progressions accompanied by systemic organ aging and age-related diseases. Both types of cellular senescence are characterized by the activation of cyclin-dependent kinase inhibitors (p53, p21, and p16), phosphorylated p38, and senescence-associated beta-galactosidase activity (Figure 1) (Kumari and Jat, 2021).

**FIGURE 2:**
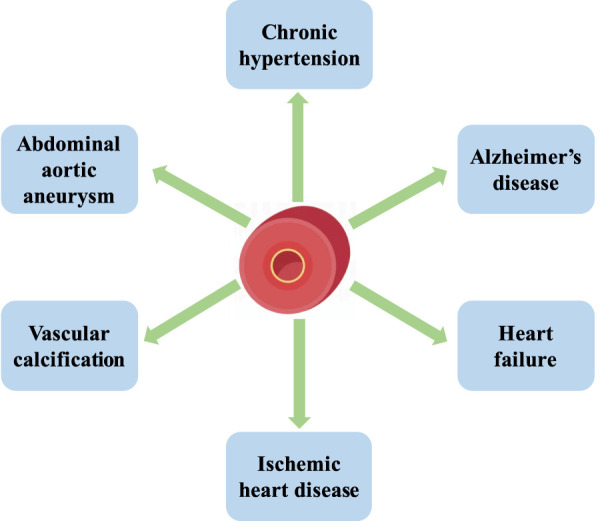
The homeostasis of vessels plays a crucial role in maintaining physical fitness and preventing cardiovascular diseases. Vascular aging causes chronic hypertension, Alzheimer’s disease, heart failure, ischemic heart disease, vascular calcification, and abdominal aortic aneurysm.

Aging is characterized by declined resting cerebral blood flow, damage in the integrity of the blood-brain barrier, and impaired vascular reactivity. Vascular aging causes structural and functional changes in the brain, damage to large and small cerebral vessels, and coronary artery calcifications, which enhance the expression of apolipoprotein E4 (ApoE4) carriers. ApoE4 carriers are the strongest genetic risk factor for Alzheimer’s disease, the major cause of cognitive disorder. Vascular aging causes arterial stiffness and an increased intima/media thickness ratio, which reduces arterial compliance and leads to chronic hypertension (Cortes-Canteli and Iadecola, 2020). It induces left ventricular pressure overload, cardiac inflammation, remodeling, and heart failure (Besse et al., 2022). Aging arteries are characterized by vascular calcification, which is associated with dyslipidemia, diabetes, and atherosclerosis. With the elevated production of p16, p21, phosphorylated p38, shortened telomere length, and telomerase activity, aging vessels increase the prevalence of ischemic heart disease, abdominal aortic aneurysm, and intimal hyperplasia (Machado-Oliveira et al., 2020). Thus, the homeostasis of vessels plays a crucial role in maintaining physical fitness and preventing cardiovascular diseases (Figure 2).

## 3 Endothelial senescence promotes aging-related CVD

The mechanisms underlying aging and aging-related CVD are complex and have not been clearly elucidated; non-etheless, cellular senescence is the core component of the pathological aging process in blood vessels. The endothelium is critical in maintaining vascular homeostasis and physiological processes such as blood coagulation, angiogenesis, blood pressure regulation by secreting active vasoactive factors. Senescent endothelial cells which are occurred during aging process or caused by ROS and chronic low-grade sterile inflammation reportedly possess prothrombotic, pro-oxidant, pro-inflammatory, and vasoconstrictor properties (Theofilis et al., 2021). The switch of ECs from the steady state to the pro-inflammatory state owing to aging is not limited to the cardiovascular system. However, it occurs throughout the whole body, causing various systematic diseases.

Several factors promote endothelial senescence. *In vitro* studies indicate that disturbance of blood flow promotes endothelial senescence, which aggravates atherosclerosis. Increased levels of hydrogen peroxide (H_2_O_2_) and O^2−^ and decreased nitric oxide (NO) production are thought to accelerate endothelial senescence (Ungvari et al., 2010). Another study demonstrated that the administration of glucose and insulin increases the production of ROS derived from the activity of the NOX2 isoform of NADPH oxidase, promoting the elevated production of p53 and cellular senescence (Fan et al., 2017). L5 is the most electronegative subfraction of low-density lipoprotein (LDL) and promotes endothelial senescence by activating the ATM/Chk2/p53 pathway (Wang et al., 2018).

Increased expression of intercellular cell adhesion molecule-1 (ICAM-1) and NOX2, decreased production of endothelial nitric oxide synthase (eNOS), and reduced NO activity is more commonly reported in replicative senescent ECs than young ECs (Minamino et al., 2002). Senescent cells are characterized by flattened and enlarged morphology, a senescence-associated secretory phenotype, and chronic sterile inflammation, including elevated expression of pro-inflammatory cytokines Interleukin-1β (IL-1β), IL-6, IL-18, tumor necrosis factor α (TNF-α), monocyte chemoattractant protein-1, C-C Motif Chemokine Ligand 19 (CCL19) and CCL21 (Ungvari et al., 2010). Senescent human aortic ECs exhibit elevated expression of vascular endothelial growth factor (VEGF) A_165_b, which is also detected at high levels in patients with coronary heart disease.

**FIGURE 3:**
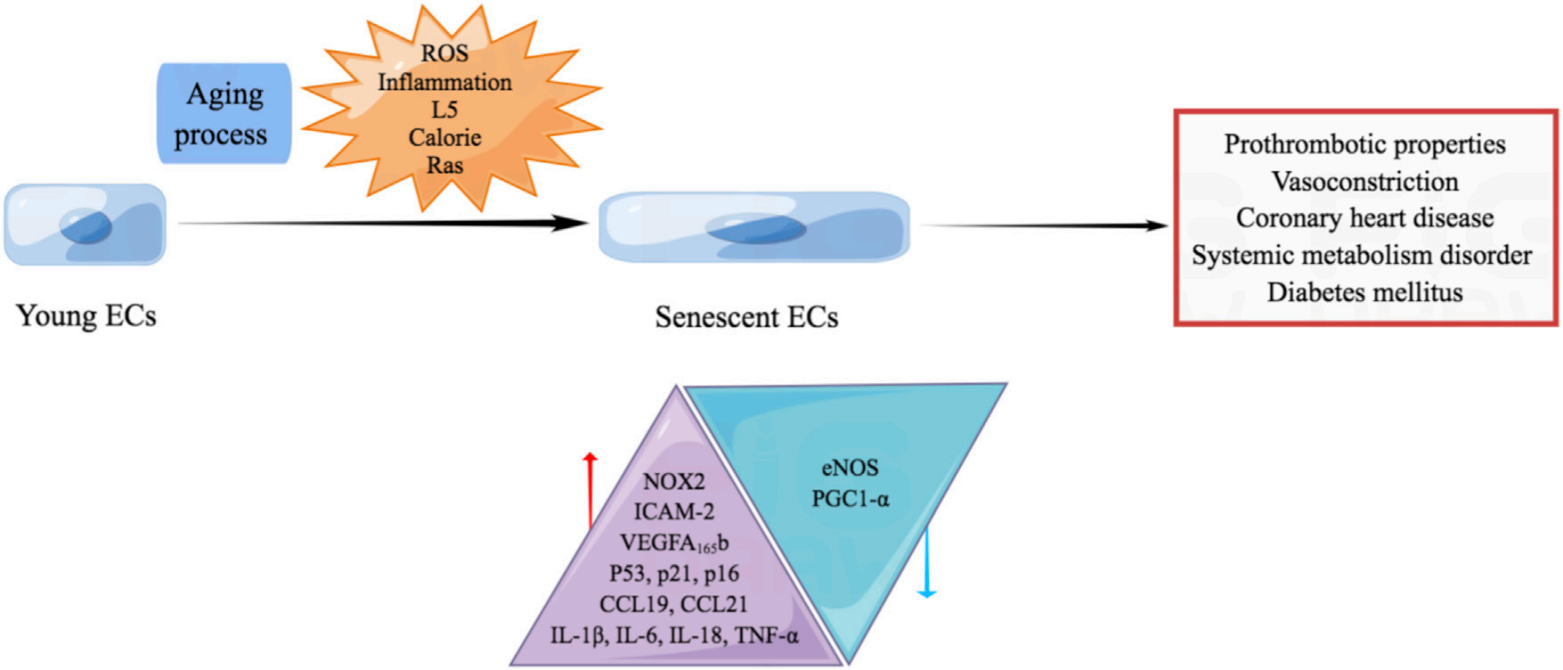
Endothelial senescence promotes aging-related CVD. Senescent ECs which are occurred during aging process or caused by ROS, chronic low-grade sterile inflammation, L5, excessive calorie and Ras are reported to possess prothrombotic, pro-oxidant, pro-inflammatory, and vasoconstrictor properties. Senescent ECs promote the occurrence of coronary heart disease, systemic metabolism disorder and diabetes mellitus. Senescent ECs exhibit increased NOX2, ICAM-2, VEGFA_165_b, p53, p21, p16, CCL19, CCL21, IL-1β, IL-6, IL-18, and TNF-α. Meanwhile, decreased eNOS and PGC1-α are observed in senescent ECs.

Senescent ECs caused by excessive calorie exhibit elevated production of p53 and downregulated expression of peroxisome proliferator-activated receptor-γ coactivator-1α (PGC1-α). The overproduced p53 upregulates the production of glucose transporter-1, which promotes glucose uptake by skeletal muscle and contributes to systemic metabolism disorder (Yokoyama et al., 2014). Senescent ECs induced by Ras cause systemic metabolic dysfunction, obesity, and diabetes mellitus by inhibiting the insulin/insulin receptor/insulin receptor substrate/phosphoinositide 3-kinase (PI3K)/Akt signaling and enhancing the insulin receptor/son of sevenless/growth factor receptor bound protein/mitogen-activated protein kinase (MAPK) signaling (Figure 3) (King et al., 2016).

## 4 Senescence of VSMCs contributes to vascular dysfunction

VSMCs play a vital role in maintaining the structural integrity of blood vessels and regulating blood pressure. Aging causes phenotype transformation and inflammation of VSMCs (Liu and Gomez, 2019). Aging VSMCs transition from the “contractile” to the “synthetic” phenotype, and the arterial media becomes thicker with aging. These structural changes are attributed to increased collagen production and decreased elastin content, which reduces the compliance of the artery, increases arterial stiffness, and contributes to higher systolic blood pressure and lower diastolic pressure (Harvey et al., 2015).

Anti-aging biochemical agents can heal atherosclerosis. The extent of VSMC senescence is correlated with the increased plaque size in atherosclerosis but not plaque formation. Senescent VSMCs from aged tissues secrete matrix-degrading proteases to induce plaque instability and increase the incidence of atherosclerosis-related complications (Grootaert et al., 2018). Free radicals are accumulated in age-associated senescent VSMCs and promote the formation of atherosclerosis because deficiency of aldehyde dehydrogenase 2 (ALDH2) and overexpression of NOX4 accelerate mitochondrial H_2_O_2_ and superoxide production (Canugovi et al., 2019; Zhu et al., 2019). The senescent VSMCs are accompanied by senescence-associated secretory phenotype, which is linked to increased production of inflammatory cytokines, including IL-1α, IL-1β, IL-6, IL-8, and TNF-α (Richardson et al., 2018). VSMCs are pivotal to arterial intimal calcification by promoting the release of extracellular and apoptotic vesicles and the production of collagen and elastin matrices (New et al., 2013). Aging VSMCs exhibit aggravated osteogenic pathways, including Runt-associated transcription factor 2, bone morphogenetic protein 2 (BMP-2), alkaline phosphatase, osteopontin (OPN), and osteoprotegerin. Decreased miR-542-3p causes BMP-7 to induce osteogenic transformation in senescent VSMCs of aging rats (Stojanovic et al., 2020).

**FIGURE 4:**
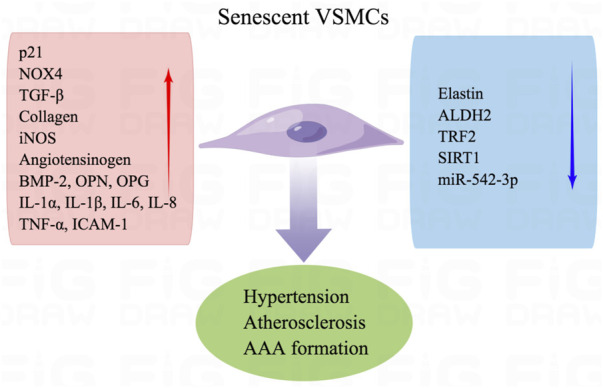
Senescence of VSMCs contributes to vascular dysfunction. Senescent VSMCs are attributed to hypertension, atherosclerosis, and AAA formation. Aging VSMCs exhibit aggravated p21, NOX4, TGF-β, collagen, iNOS, angiotensinogen, BMP-2, OPN, OPGIL-1α, IL-1β, IL-6, IL-8 TNF-α, and ICAM-1. Besides, decreased elastin, ALDH2, TRF2, SIRT1, and miR-542-3p are observed in senescent VSMCs.

Senescent VSMCs overproduce pro-inflammatory cytokines, growth factors, and extracellular matrix modifiers. Senescent VSMCs are found in atherosclerotic plaque and exhibit decreased telomeric repeat-binding factor-2 (TRF2) expression. TRF2 is a protein that localizes in telomeres and plays a critical role in maintaining telomeres, reduces DNA damage, accelerates DNA repair, and suppresses cellular senescence (Wang et al., 2015). Senescent VSMCs in aged mice demonstrate decreased expression and activity of sirtuin 1 (SIRT1) and increased p21 expression. SIRT1 activation inhibits the inflammation and senescence of VSMCs and prevents the incidence of AAA (Chen et al., 2016). SIRT1 overexpression in VSMCs inhibits AAA formation in Apoe^−/−^ mice. In angiotensin (Ang) II-induced senescence, an actin-binding protein smooth muscle 22α (SM22α) suppresses E3 ubiquitin ligase (Mdm2)-induced p53 degradation and promotes IL-6 (Miao et al., 2017). The functional and structural changes of senescent VSMCs arise from the dysregulation of transforming growth factor-β (TGF-β) signaling, enhanced expression of inducible NOS (iNOS), ICAM-1, and angiotensinogen. In replicative-senescent VSMCs, oxidative stress induces DNA damage and suppresses telomerase activity, which contributes to telomere shortening and accelerates the progress of atherosclerosis in the fibrous cap region (Figure 4) (Matthews et al., 2006).

## 5 Other types of cells in aging vessels

Adventitial fibroblasts are the primary cellular component of the vascular adventitia, which participates in the progress of inflammation and remodeling in aging. Damaged fibroblasts exhibit oversecretion of ECM and participate in vascular remodeling. ECM is regulated by matrix metalloproteinases (MMPs), whose expression and bioavailability are increased by ROS (Hua and Nair, 2015). Perivascular adipose tissue (PVAT) surrounds the vascular beds and affects vascular function by mediating paracrine signaling. PVAT regulates vascular homeostasis and contributes to vascular dysfunction in aging by releasing adipocytokines, chemokines, and growth factors. PVAT inflammation facilitates vascular oxidative stress and inflammation, increasing the risk for cardiovascular diseases. Furthermore, PVAT disorders contribute to endothelial dysfunction, neointimal formation, aneurysm, and vascular stiffness (Queiroz and Sena, 2020).

The senescence of immune cells plays a crucial role in the development of atherosclerosis (Childs et al., 2016). Individuals possessing leukocyte DNA with short telomeres, a characteristic of aging, have a higher mortality rate than the young population. Senescent monocytes tend to exhibit increased ROS and pro-inflammatory cytokines, such as monocyte chemoattractant protein-1 (MCP-1), IL-6, IL-1β, and TNF-α, and induce intimal foam cell accumulation to drive the formation of atherosclerotic plaques. Genetic or pharmacological deletion of senescent monocytes disrupts atherosclerosis progression. p16^INK4a^ promotes inflammation and induces macrophage senescence, which accelerates plaque formation in atherosclerosis. However, the deletion of p16^INK4a^-positive cells leads to reduced cardiomyocyte cross-sectional area (Owens et al., 2021). The number of senescent capillaries is reduced in metabolically active organs, such as skeletal muscle and brown adipose tissue, with lower systemic insulin sensitivity (Prior et al., 2015). The discovery of senolytic compounds would be a vital topic in anti-aging and anti-CVD research.

## 6 Exercise improves physical health in young and old

The benefits of exercise on the cardiovascular system have been well documented in basic research and clinical studies. Exercise reduces the incidence of atherogenesis, high blood pressure, diabetes mellitus, stroke, resting heart rate, and cardiac hypertrophy. Furthermore, it improves the plasma lipoprotein profile and increases insulin sensitivity and cardiac output (Luan et al., 2019). Exercise improves glycemic control, insulin sensitivity, and fatty acid uptake in adipose tissue, skeletal muscle cells, and endothelium in patients with diabetes mellitus (Olver et al., 2017). In summary, physical activity is widely accepted as an effective therapy in the treatment and prevention of diseases.

Mice that underwent voluntary wheel running for 6 weeks exhibited decreased levels of vascular lipid peroxidation, superoxide, endothelial dysfunction, and lesion formation (Laufs et al., 2005). Regular physical activity reduces the recruitment of inflammatory cells and the integrity of aortic valve endothelial in degenerative aortic valve disease models (Matsumoto et al., 2010). Shear forces, NO, prostacyclin, and metabolites released from active skeletal muscle during exercise progress improve vascular structure and function (Nystoriak and Bhatnagar, 2018). Physical activity reverses the age-related decline of NO bioavailability and elevation of ROS, p53, p21, and p16 levels, as well as NOX activity, indicating that exercise inhibits cellular senescence (Rossman et al., 2017). Shear stress induced by exercise also activates eNOS activity and NO production, in addition to preserving endothelial function. Many types of growth factors, including insulin-like growth factor 1 (IGF1), platelet-derived growth factor (PDGF), and VEGF, are induced by exercise and play crucial roles in maintaining vascular function (Bernardo et al., 2018).

Physical activity induces the mobilization of endothelial progenitor cells (EPCs), which play a crucial role in endothelial repair and angiogenesis and counteract endothelial dysfunction. In one clinical trial, 12 weeks of supervised exercise training (SET) improved endothelial health by increasing the number of circulating EPCs, improving flow-mediated dilation of the brachial artery, and decreasing the plasma levels of asymmetric dimethylarginine in patients with coronary artery disease (Steiner et al., 2005; Schlager et al., 2011). Regular ET also increases the number and migration capacity of circulating progenitor cells, increases the VO_2_ max, and decreases the level of brain natriuretic peptide, thereby protecting against vascular disorder by targeting EPCs (Brehm et al., 2009). A two-year study showed that a high-intensity exercise program preserved the cardiac ejection fraction, decreased left ventricular stiffness, and reduced the incidence of heart failure in middle-aged populations. Even aged groups can benefit from the functional and structural changes to the cardiovascular system elicited by physical activity (Howden et al., 2018).

## 7 Exercise preserves senescent vascular function

Compared with the sedentary population, exercising adults do not show age-induced elevation of p53, p21, or p16 in old age. Clinical studies suggest that habitual aerobic exercise prevents oxidative and inflammatory signaling in ECs and preserves brachial artery flow-mediated dilation in aging adults (Rossman et al., 2017). Exercise counteracts endothelial senescence through anti-oxidative and anti-inflammatory mechanisms and by reducing lipid peroxidation (Golbidi and Laher, 2013). An exercise-promoted anti-oxidative effect may be evoked by the adaptation of ROS and compensation of repair mechanisms for oxidative damage, which lower the ROS levels and increase oxidative stress resistance in aging muscle and endothelium (Kozakova and Palombo, 2021). Heme oxygenase-1 (HO-1), induced by the Nrf2-Keap1 signaling pathway, promotes ferritin synthesis, diminishes the cellular pool of free iron, and enhances the levels of potent antioxidants and bilirubin which is increased in endurance-trained men to recover the senescent red blood cells (Kerins and Ooi, 2018). However, baseline HO-1 expression is downregulated in athletes as an adaptive mechanism for regular exercise (Niess et al., 1999).

Exercise produces a short-term pro-inflammatory response followed by a long-term anti-inflammatory response (Kasapis and Thompson, 2005). IL-6 is the first cytokine released from contracting skeletal muscles to the circulatory system during the initiation of exercise (Erdei et al., 2007). IL-6 acts as a pro-inflammatory cytokine that stimulates the immune response and an anti-inflammatory cytokine that inhibits TNF-α and IL-1β and activates IL-10 (Keller et al., 2001; Nieman et al., 2003; Febbraio and Pedersen, 2005; Nielsen et al., 2007). However, physical exercise prevents the elevation of systemic inflammatory molecules, including IL-6, TNF-α and CRP in middle aged and older people (Zheng et al., 2019). Besides the reduction of pro-inflammatory cytokines, physical activity also increases the expression of anti-inflammatory mediators, IL-10, IL-15 and adiponectin, attenuates apoptotic DNA fragmentation, and downregulates TNF-α-induced apoptotic pathways in skeletal muscles (Figueras et al., 2004). Exercise increases the production of follicle-stimulating hormone 1 (Fstl1), which improves the repair of ECs and inhibits the production of inflammatory cytokines in the elderly (Miyabe et al., 2014; Abd El-Kader and Al-Shreef, 2018). In aging rat models, aerobic exercise training could improve endothelial dysfunction by inhibiting the production of inflammatory factors, including IL-6 and TNF-α, and elevating SIRT1 expression and eNOS activity (Gao et al., 2022). Moderate and intense physical activities augment laminar flow and eNOS expression, activate inward rectifying K^+^ channels, drive membrane hyperpolarization, and promote the uptake of extracellular Ca^2+^ into cells. Elevated intracellular calcium levels cause the dissociation of caveolae-bound eNOS and NO synthesis (Johnson et al., 2011). Increased NO synthesis amplifies the shear stress-induced expression of superoxide dismutase (SOD) to inhibit ROS-induced degradation of NO (Gielen et al., 2010). Long-term aerobic exercise promotes the IGF-1/PI3K/p-Akt signaling pathway to stimulate the expression of eNOS and alleviate apoptosis and inflammation during aging (Lin et al., 2020). In contrast, high levels of ROS induced by exercise can rapidly be converted to H_2_O_2_ by SOD1 and SOD3, which further increases the expression of eNOS in endothelium (Drummond et al., 2000; Cai et al., 2001). Besides the direct protective role of eNOS, it also regulates telomere reverse transcriptase (TERT) expression in endothelium (Werner et al., 2009). TERT and TRFs constitute the main component of the telomere complex located at the ends of eukaryotic chromosomes and prevent the genome from degradation (van Steensel et al., 1998). In the exercise group, the increased expression of telomerase and TERT regulate cell growth and survival in aorta, as well as elicit an anti-apoptotic role in endothelium. Exercise upregulates the expression of TRF1 and TRF2 and regulates clonogenic potential, migratory capacity, cellular aging, and function. The expression of Chk2, a DNA damage checkpoint kinase, is also upregulated by exercise training accompanied by increased telomerase expression in endothelium (Werner et al., 2009).

**FIGURE 5:**
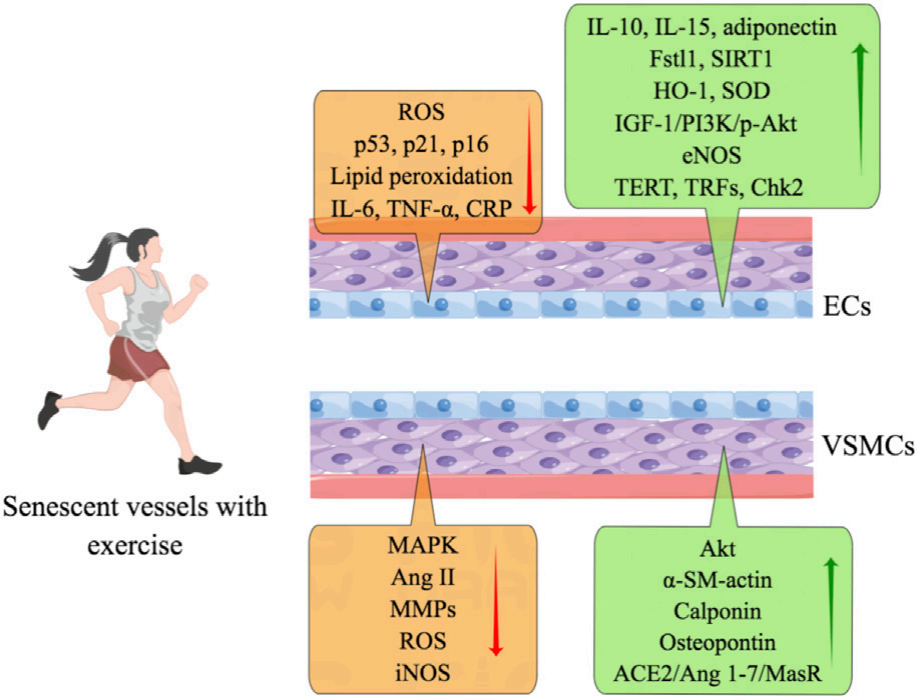
Exercise preserves senescent vascular function. Exercise counteracts endothelial senescence through anti-oxidative and anti-inflammatory mechanisms and by reducing lipid peroxidation. Physical activity also increases the expression of IL-10, IL-15, adiponectin, Fstl1, SIRT1, HO-1, SOD, IGF-1/PI3K/p-Akt, eNOS, TERT, TRFs, and Chk2. Physical exercise improves the function of senescent VSMCs by inhibiting the expression of MAPK, Ang II, MMPs, ROS, and iNOS. And exercise facilitates the productions of Akt, α-SM-actin, calponin, osteopontin, and ACE2/Ang 1-7/MasR.

Physical exercise improves vascular function in vascular aging and diseases by suppressing the phenotype switching of VSMCs (Zha et al., 2022). Exercise facilitates the VSMCs maintaining the contractile phenotype and reversing expression of differentiation proteins, including α-SM-actin, calponin, and osteopontin, by activating Akt, inhibiting the MAPK signaling pathway, and suppressing the thickening of blood vessel walls (Zhang et al., 2019). In a meta-analysis, the average reduction of 7 mmHg in systolic blood pressure (SBP) and 5 mmHg in diastolic blood pressure (DBP) were observed in patients with hypertension who regularly exercise compared with that in sedentary patients with hypertension. In the normotensive population, exercise reduces 3–5 mmHg in SBP and 2–3 mmHg in DBP (Cornelissen and Fagard, 2005). What is more, the reduction of blood pressure is observed in elderly hypertensive patients associated with a decrease in stroke volume (Brandao Rondon et al., 2002). The RAS system is shifted from the angiotensin converting enzyme 1/Ang II/AT1R to the protective angiotensin converting enzyme 2/Ang 1-7/MasR axis by physical activity in VSMCs of the hypertension model (Figure 5) (Frantz et al., 2017).

Exercise decreases the expression of MMPs to regulate the synthesis and degradation of ECM in aging, including collagen and fibronectin, by inhibiting the production of ROS (Zhang et al., 2016). One study showed a reduction of MMP-9 levels was found in an elderly female population (62–73 years old) when they participate an exercise program (Fiotti et al., 2009). In Guzzoni’s work, they reported increases of load carried contributed to reduce collagen accumulation within the left ventricle which was associated with higher MMP-2 activity and TIMP-1 expression (Guzzoni et al., 2017). Exercise changes the metabolic phenotype of PVAT, stimulates angiogenesis in adipose tissue, inhibits the expression of inflammatory molecules and iNOS production, and improves the activation of eNOS and the expression of Mn-SOD, which preserves vascular reactivity (Huang et al., 2018). Aerobic exercise decreases leptin levels in PVAT, improves leptin resistance, alleviates hypoxia and macrophage infiltration, and prevents infiltration of immune cells into PVAT, which further prevents vascular dysfunction in age-induced obesity (Pedrinolla et al., 2018). Physical activity also has a significant therapeutic effect on aging women. In aging women, aerobic exercise significantly reduces the levels of endothelin 1 and calcium, rescues the impairment of NO synthesis, preserves the plasma concentration of cGMP and endothelial function, and is more effective than estrogen replacement therapy (Park et al., 2008).

## 8 Impact of different exercise modalities

Although the beneficial effects are clear, the mechanisms and optimal frequency of exercise for promoting cardiovascular health remain unknown. Exercise intensity and pattern influence the cardioprotective effect of aerobic exercise. The appropriate exercise intensity for promoting cardiovascular health refers to 60% of the maximal heart rate (Mezzani et al., 2013). A moderate level of exercise is consistently associated with the reduction of cardiovascular disease risk, but continuous high-intensity exercise has detrimental effects on cardiovascular health. The impaired myocardium and elevated cardiac troponin I and troponin T levels which reflect myocardial cell damage are observed after vigorous prolonged exercise (Thygesen et al., 2012). The injured cardiac function, including reduced contractility and right ventricular and left ventricular ejection fraction are reported after long periods of high-intensity training (Elliott and La Gerche, 2015). What is more, elevated troponin levels are found even in the well-trained athletes (Kleiven et al., 2020). Although there appears to be a dose-response relationship between higher intensity activities and greater health benefits in older people, higher risks of adverse events are accompanied with high-intensity activities (Kim et al., 2013). Therefore, high-intensity training is not advisable for older people and CVD patients because of the higher risk of cardiac accidents than that of moderate-intensity training (Dun et al., 2019).

In a clinical trial, dyslipidemic individuals were divided into high exercise frequency/high intensity (23 kcal/kg/week, jogging), low exercise frequency/high intensity (14 kcal/kg/week, jogging), and low frequency/moderate intensity (14 kcal/kg/week, walking) groups for 6 months; there was a dose-dependent effect of exercise frequency on lowering plasma levels of LDL, triglycerides, very low-density lipoprotein (Kraus et al., 2002). In a longitudinal analysis, significant improvements in physical and mental function are associated with increased frequency of exercise among the participants (aged 65 and older) from SilverSneakers fitness program (a nationwide fitness program for older adults). In the peripheral artery disease (PAD) and high cholesterol mice model, voluntary training resulted in a higher running speed and a greater total distance, leading to a better training effect than forced treadmill walking (Bresler et al., 2019). Forced treadmill running exacerbates, whereas voluntary wheel running reduces colitis symptoms and colon inflammation in a mouse model of colitis (Cook et al., 2013). The discrepancy in the exercise-training effect might be caused by the difference between the innate drive of the individual for voluntary running and the distress and depressive behavior of individuals subjected to forced treadmill running. High-intensity interval training (HIIT) appears to elicit a stronger positive effect than moderate exercise on maximal aerobic capacity and the VO_2_ peak, which is recognized as an indicator of good prognosis in CVD (Ito, 2019). Referring to the tolerability and effects of HIIT in senior population, various researches of HIIT are conducted. 28 weeks of HIIT in older adults (with a mean age of 65 years or older) is found to elicit decreased systolic and diastolic blood pressure proving that HIIT protocols are generally well-tolerated in aging population (Reichert et al., 2016). Discrepancies that whether HIIT attenuates, increases, or has no impact on flow-mediated dilation (FMD) are observed in aging adults depending on sex and cohort’s fitness (Currie et al., 2012; Bailey et al., 2018). Much variation of frequency and duration exists in HIIT. Although some HIIT protocols appear more commonly used, there is still no consensus on which HIIT protocol is most likely to induce training results in older population and further research is needed about the protocols of HIIT.

Different exercise modalities bring different therapeutic effects in older (>50 years) adults. Based on the increased vascular stiffness and impaired body adaption in older adults, resistance training seems less effective at improving cardiac autonomic control in the aging population compared with that in the young population. Acute resistance training increases the heart rate and blood pressure in a short period, which may be harmful to the cardiovascular system. Souza et al. (2017), observed an increase in aortic wall thickness, elastic lamina and collagen fibers in chronic resistance training using the climbing vertical ladder model (Grans et al., 2014). However, this long-term resistance training model prevents the increase in mean arterial pressure, sympathetic modulation and impaired baroreflex sensitivity (Speretta et al., 2016). On the other hand, using the jumping in the water tank with the load apparatus attached to the tail model to mimic the chronic resistance training model did not reverse changes in cardiac function (Lima et al., 2015). Therefore, resistance training should be carefully evaluated before it is performed by older adults (Martinek et al., 1972). Compared to resistance training, endurance training induces a more marked decrease in blood pressure, which suggests a better therapeutic effect of endurance training (Wanderley et al., 2013). In some studies, the extent of improvement in vascular function by physical activity depends on the duration of exercise. Long periods of exercise (1 h/day on the treadmill, 5 days a week, for 8 weeks) induced the relieved aortic contraction response to noradrenaline and preserved relaxation caused by acetylcholine. However, these changes were not observed in the accumulated exercise group (four periods of 15 min per day on the treadmill, 5 days a week, 8 weeks) (Pagan et al., 2018). Compared with traditional physical activity patterns, coordinative training such as Tai Chi and dancing offer a more suitable training modality for older adults, with the advantages of greater enjoyment, interactivity, tolerance, and fewer safety concerns for the exerciser (Rosen et al., 2011).

## 9 Conclusion

Integrity of endothelial and vascular smooth muscle cells is essential to vascular homeostasis and to prevent CVD. Vascular cell senescence is the core of the pathological process in aging blood vessels and causes several diseases, including CVD. It is widely accepted that exercise programs have beneficial effects on CVD patients by improving cardiovascular function, quality of life, and cardiorespiratory fitness. Exercise protects senescent cells from dysfunction by preventing telomere shortening, cell cycle arrest, and the overproduction of inflammatory molecules. Both men and women can benefit from exercise to counteract the CVD, even at an advanced age. However, further research is required to elucidate the optimal intensity and modality for protecting the cardiovascular system.
